# Feminist affects of folds, death and dirt in the photobook *Reconstrucción* by Rosana Simonassi

**DOI:** 10.1080/17540763.2025.2484392

**Published:** 2025-06-05

**Authors:** Briony Carlin

## Abstract

Reconstrucción, 2016, by Rosana Simonassi (b.1974), is a materially complex photobook that critiques the media’s fascination with fatal acts of violence against women, while destabilising the relation of photobook, reader and archive. Close analysis of a specific encounter with Reconstrucción in the National Art Library in 2018 foregrounds three aspects of the book — folds, death and dirt — which emphasise realities of its circulation and consumption. While Reconstrucción ostensibly comprises restaged scenes of femicide, its use of obstructing pages, unusual folds and unexpected spills of dirt deploys the material form of the photobook to generate a more reflexive, implicated readerly encounter with the spectacle of death. By situating the photobook in relation to the artist’s wider practice, the article argues the photobook offers a new logic for presentation and dissemination that: expands the artwork’s conceptual resonance; re-examines photography’s relationship with death; disrupts notions of authorship and readership; and speaks broadly to issues of gendered violence. These insights also reveal tensions around how photobooks are accessed and encountered in institutional spaces, virtually through databases, and in reading rooms. The epistemological regime of the museum art library thus makes ontological understandings about this individual photobook and the historiography of the medium more generally.

## Introduction

A photobook is a tactile, imaginative experience, which stages an encounter between reader and object, situated in the world. Looking at a photobook about murdered women, in the sanitised, hierarchised and patriarchal space of the art museum library, is therefore a deeply complex encounter.

This article speaks about one such encounter with *Reconstrucción*, by Rosana Simonassi (b.1974). In what follows, I activate a situated encounter with this photobook, read in the National Art Library (NAL) at the Victoria and Albert Museum (V&A) in 2018, to critique the realities of photobook circulation and consumption in institutional collections, whilst expanding on recent scholarship around intersections between feminism, photography and gendered violence.

Rosana Simonassi is an Argentinian artist whose visual practice negotiates between aesthetic theory, visual culture and philosophy. In *Reconstrucción*, the artist posed her body in scenes reconstructed from public domain crime scene photographs of femicide. Simonassi’s photographs were created against the backdrop of La Marea Verde, or ‘Green Wave’ of feminist protests in Latin America that swelled around abortion rights. Her project critiques society’s representation of fatal acts of violence against women, whilst seeking to synthesise the media’s fixation with the female corpse-body with photography’s historical relation to death.

**Fig. 1.**
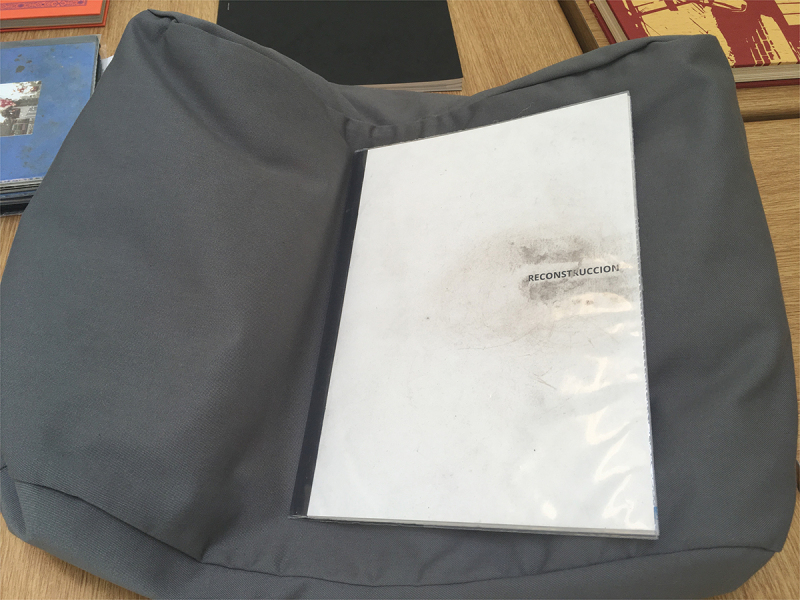
Cover of *Reconstrucción* by Rosana Simonassi, as seen in the national art library, positioned on a book cushion. This copy has an archival CoLibri wrapper and encased dirt. Object information: NAL call number 808.AT.0008; museum no. 38041017000407; acquisition supported by the American friends of the V&A through the generosity of David Solo. Photo: Briony Carlin.

**Figs. 2–5.**
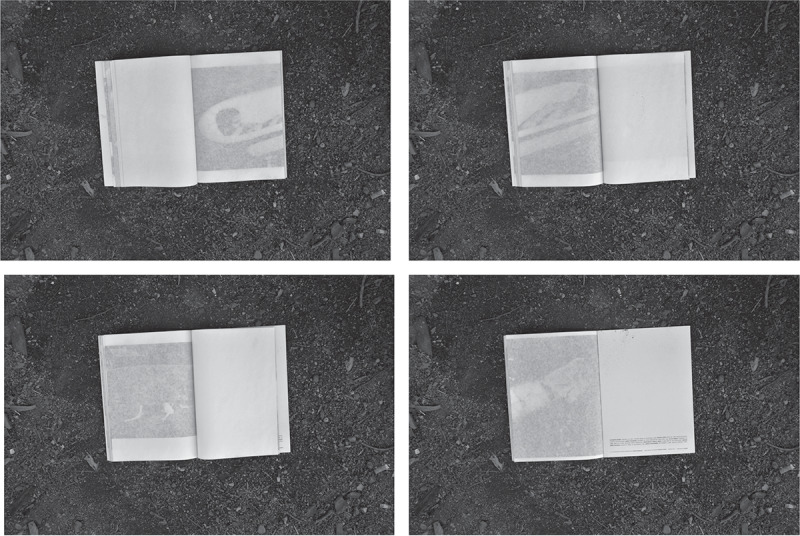
Page spreads from *Reconstrucción*. photo: ©Rosana Simonassi, all images reproduced with permission from the artist.

*Reconstrucción* was designed and published by Verónica Fieiras, via her Spanish Editorial Chaco, which showcases ‘Latin American authors who use the image as a space for reflection, to throw uncomfortable and sharp projects into the world’.^1^ In the photobook, the photographs are partially hidden by translucent sheets of paper that are wrapped around each page and sewn into the binding. The book is packaged in an envelope filled with dirt (Figures 1 and 2). *Reconstrucción* employs these obstructing pages and unexpected spills of dusty particles to provoke complicity, protection, voyeurism and shame in relation to the topic of femicide.

There is a growing current of scholarship on the feminist potentials of the photobook, focussing on the photobook’s production, distribution and stakeholders, its varying social and economic functions, and makers of marginalised identities.^2^ As Annelys de Vet has noted, ‘the future of the book is not about the book, but about its layered sensorial ways of connecting, its common way of capturing narratives and its pluriversal distribution.’^3^ This is a welcome departure from the majority of criticism before the 2010s, which often served to reinforce the extant photo historical canon and tended towards anthology accounts of mostly male photographers comprising region-specific surveys and Anglo-European histories.^4^ In this earlier discourse, photobooks were treated as singular productions authored by the photographer, which primarily served the production of a collector’s market.^5^ Feminist art historian and photography publisher Delphine Bedel has re-oriented a conceptualisation of the photobook away from its nature as an object or product, towards an ‘artist’s publishing practice’.^6^ Importantly, this emphasises the processual and collaborative nature of making photobooks. As Rebecca Brown has identified in relation to Dayanita Singh’s work, the photobook supports opportunities for intimacy between an artist and their subject, and a rearticulation of the archive, as part of an ongoing feminist praxis.^7^ As with other artists’ publishing practices, creative practitioners have often turned to the photobook medium as a form of activism; however, with less imperative for distribution or engaging with market practices, there has been comparatively less criticism that engages with *how* these visual-material objects function to move their readers towards ideological or political change.^8^ To this, I would add that we also need a processual and contingent understanding of where and how photobooks are structured and consumed in order to envisage their wider socio-political potentials.^9^

This article makes theoretical and methodological contributions to this objective through tying together the verbal, artistic practice and socio-political aspects of how one copy of a photobook can perform as an object in the world. Scrutiny of photobooks in collections is needed because the photobook’s invitation to be handled is antithetical to the visual regime and modernist gaze prescribed by the art museum that has historically been designed for its ideal majority-culture visitor. More than establishing an art historical positioning for one particular artwork or artist, my aim is to show critical insights that emerge from analysing situated photobook encounters. In this case, the encounter with *Reconstrucción* is activated to think critically about feminism, experience and institutional hierarchies of knowledge. Photobooks are only partially visual objects, and their critique requires more tools to engage with embodiment and affect than traditionally employed in the discourse of photography. In this context, I employ Margaret Wetherell’s understanding of affect as ‘embodied meaning-making’, or a pre-verbal ‘intensity’ of experience, which becomes identified and fixed as linguistically coded emotion.^10^

The research is organised around three concepts of folds, death and dirt. These formal elements of *Reconstrucción* stage an affective encounter with the spectacle of death and can be leveraged to reveal formal, conceptual and institutional modes by which the bodies of women are disciplined. In my analysis, the translucent folded pages frustrate readers’ access to the images and draws attention to a more self-conscious reading encounter, in which the bodies of women are structured within the pages of two different photobooks with folded pages, meanwhile my own reader-body is directed towards certain behaviours. The topic of death opens up discussion of the representation of real occurrences of gender-based violence with sensitivity for the associated victims, whilst connecting with photo-historical concepts around corporeality, subjectivity and authorship in photography. As co-produced objects, photobooks destabilise traditional roles of ‘author’ and ‘reader.’^11^ This links with feminist notions of more symmetrical collaboration, in opposition to the hierarchical and capitalist modes of working. Finally, I engage both the reality of dirt and the metaphor of errant matter to analyse how *Reconstrucción* destabilises the ‘purifying’ epistemological regime of the museum art library. The analysis situates the photobook encounter as a discursive event through revealing differential, performed meanings about photobooks as multiple, relational ‘boundary objects’.^12^

Theoretically, the analysis follows a feminist epistemological ethic drawing from Donna Haraway’s theorising on situated knowledges; theories of affect and atmosphere advanced by Sara Ahmed and Shanti Sumartojo, and Erin Manning’s writings on the minor; and feminist new materialism; all of which propose alternative modes of attuning to and moving through the world as a challenge to the dominant neurotypical, imperialised, anthropocentric and heteronormative structuring of human experience.^13^

I narrate the research in first person to underline the multiple, personal acts of meaning-making that contribute to understandings of artist’s printed matter, and to identify non-human agency in structuring these insights. As Haraway puts it: ‘Situated knowledges require that the object of knowledge be pictured as an actor or agent’.^14^ Zdenka Badovinac argues situatedness is essential to thinking about curatorial discourses which necessarily account for ways of experiencing individual environments.^15^ To this end, my research uses auto-ethnography as a method of ‘epistemic generosity’, which open up minor registers of looking, sensing, feeling, knowing, comparing.^16^ To this primary data, multiple layers of reading, interview and secondary literature have been applied to construct the analytical framework. The research is informed by conversations with Rosana Simonassi in December 2022 and Jennifer Reeves in 2018–2019, who was at that time Librarian, Collections Section NAL.^17^

## Into the Fold: situating a photobook encounter

**Figs. 6–7.**
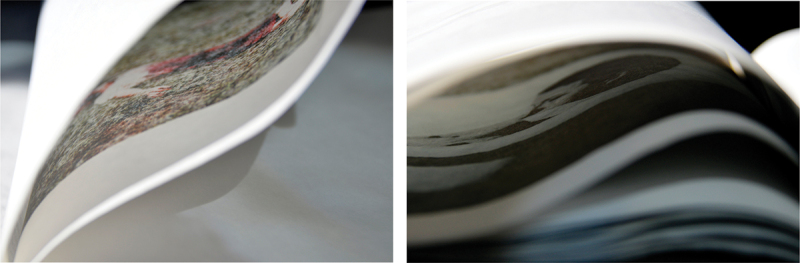
*Reconstrucción* (Fold detail). There is a translucent sheet of paper that wraps around each photograph. Photo: ©Rosana Simonassi.

I first encountered *Reconstrucción* in 2017, in the NAL, whilst working as Assistant Curator of Photographs at the V&A. The slim, clinical, dossier-like book is wrapped in plastic with encased dirt (Figure 1). It contains 7 photographs that splay around the end of one page and onto the next, partially obscured by the French fold construction (Figures 2–7). When I requested to see the book a second time, the librarian suggested another title using the same design solution. The specific juxtaposition between materially similar books, in the public arena of the art museum library, amplified my personal knowledge-making about the gender-based violence signified by *Reconstrucción*.

In *Reconstrucción*, the fold preserves the privacy of the (performed) victim and interrogates the public’s (via the reader’s) gruesome curiosity about the murders of young women. Simonassi has reported this structure was intended to ‘highlight the morbid tension that makes us both willing and unable to watch’, forcing the viewer to acknowledge their thirst for the actual images of corpses. However, Fieiras’s shrouded design formed a barrier between the bodies being referenced, Simonassi’s posed body, and my body perceiving the work, signifying death as an absolute separation, and questioning the extent to which we can connect with death as a concept or an image (more on this later). This overall aesthetic effect and materiality of *Reconstrucción* engages the reader in a destabilising reading experience that thwarts their anticipation of seeing images. To tilt the book and glance between the stiff crease would be awkward gesture that could damage the paper. The form therefore enacts a trial of voyeurism. This performative ambiguity distinguishes *Reconstrucción* from other research-led photobooks combining images and text to investigate gender-based violence, most notably Laia Abril’s *On Rape* (2022). The form of *Reconstrucción* more closely aligns with other feminist artists’ books by materially emphasising the violable boundaries of subjectivity, self and body. For example, a potentially destructive mechanism was deployed in Suzanne Lacy’s *Rape Is*. (1972), in which readers must restage a gesture of intrusion by breaking a sticker seal on the front of this slim book detailing the emotional trauma and structural failures around sexual abuse.

That specific event of reading *Reconstrucción* in the NAL was made more memorable through the encounter with another book of folds, *Illa*, by Salva Lopez (2015). The subject matter is the author’s breakup with his girlfriend, following which he visited Lanzarote. Stark, monochrome photographs of architecture and volcanic landscape cover opaque white pages. Enclosed in the interior fold are colour photographs of his former partner, from happier times. Regardless of the maker’s intention, seen alongside *Reconstrucción*, the gesture of a man confining images of a woman within the folded pages of book felt like an uncomfortable aggression and a violation of privacy.^18^ By comparison, I interpreted the fold in *Reconstrucción* as more fiercely protective of women’s bodies and experiences. While both books were produced with an economic objective, *Reconstrucción* makes a political point that could be accusatory. If you were to look inside the fold, the sensational appetite for true crime may not be satisfied, as the images are removed from their gruesome context. If the reader truly desires to see what happened, they must reconstruct the crime in their imagination, implicating them in an act of violence that, though mental, renders them complicit. In both examples, the enclosing paper enacted simple, powerful agency upon the bodies of women represented within, acting variously to conceal, protect and confine. My interpretation of each book acted upon the other, as they became entangled in co-constituting and consolidating the meanings I made about each work, showing that responses to individual photobooks can be co-configured by other recent images, ideas or experiences.

*Reconstrucción*’s folds also affected the book’s general feel and dimension. I reflected in my journal, as I was looking up bibliographic information:

7 black and white photographs; it’s over quickly. I remember sitting there, turning the pages, slowly, and having a strange sensation of unexpected loss — because I expected it to be longer: because photobooks are generally longer, and because the folded mass of paper means the thickness of the book belies its brevity.

This slippage between the volume of pages perceived and number of pages received has a poetic resonance of being cut short, a life, for example. The sense of loss is then amplified, because although they are reconstructions, these images represent lives lost as victims of violence. I wrote the book is ‘over quickly’ – as one might describe a death.

A feeling of transience pervaded that second, more attentive reading because the 18-page book was somehow emptier than I had remembered it. Simonassi told me she wanted to recreate the way that society ‘forgets’ murders of women. While the book rematerialises, historicises and memorialises these scenes of death, simultaneously, the loss of detail in the obscured images rehearses a kind of forgetting.

The self-conscious reading behaviours invited by the folded pages drew attention to the specific moment of reading, situated within the NAL. The library collection made it possible to look at several books I did not own, gathered from different parts of the world, and which produced more nuanced, interacting responses. However, the NAL has rules and expectations that regulate a reader’s body and heighten their experience. Since Carol Duncan’s theorising on the ‘civilising rituals’ of museum spaces, scholars have reflected on the architecture and surveillance that discipline museum publics and the choreographed viewing and handling performed by users.^19^ This constitutes an affective atmosphere that is felt differently by readers of diverse backgrounds.^20^ Institutions play a considerable role in photobook circulation and consumption, yet many readers may feel uncomfortable in institutional environments, particularly those of marginalised identities, or who have not been represented by institutional agendas that are historically patriarchal, racist, ableist and classist.^21^

**Fig. 8.**
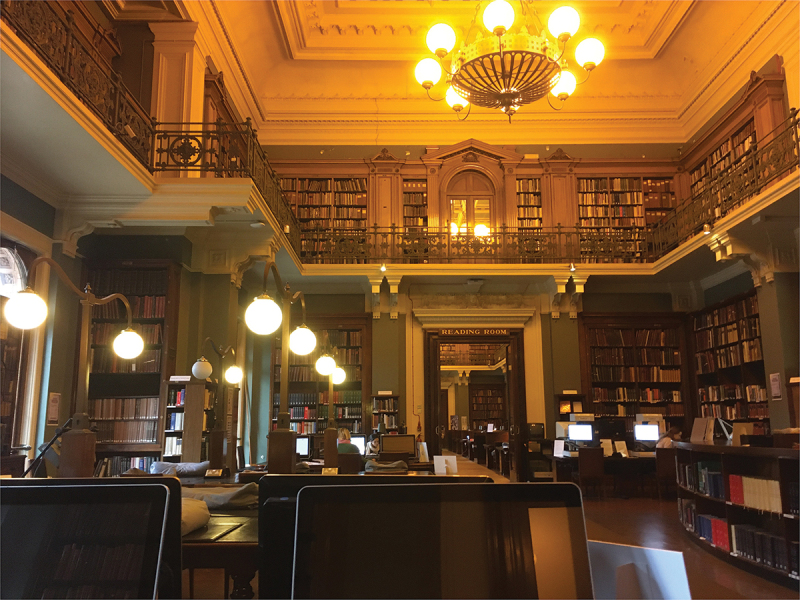
Interior of the National Art Library, with the librarian’s counter seen to the right. Photo: Briony Carlin.

The NAL is free to access, but to enter it and see a special collection, aspiring readers must navigate their (less user-friendly) online catalogue, register for an account and request material beforehand. Upon arrival at the V&A, to reach the NAL, visitors must grapple with a cloakroom, a transparent plastic bag for belongings, and an idiosyncratic floorplan to find the library on the first floor. The V&A is situated in South Kensington in London, the richest borough of the most expensive city in the UK. There are therefore many socio-economic barriers impeding a wider audience’s physical and intellectual access to the NAL and its photobook collections.

As a former member of museum staff, I was more comfortable than most in the NAL. I sat upright and supervised at a heavy mahogany table with the book placed on a cushion (Figure 8). The scripted ways of looking and page-turning in the NAL discourage more unusual ways of forming lived and sensory knowledges with objects, nevertheless, I embodied the posture and attentiveness of a studious reader in a public place, and consequently paid closer attention to the affects these books were registering in my body. The heightened awareness of the readerly encounter as invited by *Reconstrucción* emphasises necessary questions around how the bodies of women are both represented and controlled, in the media, in artworks and in institutional public space.

*Reconstrucción* exists in the NAL, and Simonassi’s work is represented in a UK institutional collection, because the book was researched and purchased by librarian Reeves. However, the photographs in *Reconstrucción* were not conceived with a book as their outcome. The next section turns to situate the photobook in relation to the earlier presentation and exhibition of the project, to consider how the format of the photobook has offered opportunities for expanding its conceptual aims as well as its circulation.

## Reconstructing death and the Author

The affective sense of ‘forgetting’, transience and voyeurism remarked in the NAL encounter expands on Simonassi’s earlier iteration of *Reconstrucción* as a print series. The project was initially a series of low-resolution photographs made on an 8-megapixel camera, printed large scale on light 70gsm billboard paper, which bled through a shadow of the image on the reverse. They were exhibited in galleries, hung back to front and taped to the wall, where they rippled with passing currents of air, and viewers had to lift the fragile object to look behind it at the ‘real’ picture.

What these ‘real’ pictures contained were images of death or an appearance of death performed by a living body. Simonassi’s series was inspired by her seeing an image of the corpse of Marilyn Monroe in the Sunday papers, in a promotion for a book by a celebrated autopsy doctor. Simonassi was captivated by how the image had been commodified. She began researching women who had become known for their deaths, compiling folders of information and becoming obsessive about the case details. She restaged images that had circulated widely in the public domain, removing the blood, cuts and scratches, shoes of police and forensic workers, and evidence markers. The imbalanced compositions mimic the framing of the ‘overall’ shots made by crime scene photographers, which as an industry conforms to very different, instrumental aims.^22^

Following an unsuccessful attempt with medium format, Simonassi relied on tools and materials to recreate what she called the discomforting ‘sordidness’ and ‘loneliness’ that constituted the ‘feeling of death’ of the original forensic images, which circulated in newspapers and were consumed over breakfast. Compared to the grotesque luxury of Sally Mann’s fine art photographic series *Body Farm*, printed in sumptuous silver gelatine, Simonassi’s method recalls the grainy halftone of a newspaper print and exaggerates the callous attitude of the media towards these violent deaths. Simonassi’s photographs, available for purchase, signified the same commodification of bodies and deaths of women that appalled and compelled Simonassi when she encountered Monroe’s deathbed in the newspaper. At a time of frequent demonstrations around abortion rights and gender-based violence in Argentina, the ephemeral material of the prints suggested how little the lives of the women were valued, in comparison to the more famous deaths.^23^

This project at large connects with a legacy of theoretical enquiry into the relationship between photography and death. In *Camera Lucida*, Roland Barthes aligns the photograph with death because it establishes a permanent and irreversible relation to *what has been*. In his discussion of a photograph of Lewis Payne in *Camera Lucida*, he identifies the image’s punctum as the realisation that ‘he is going to die, and he is dead’.^24^ However, the impact of Payne’s portrait depends upon the photograph’s veracity, something other photographers have repeatedly challenged since its invention. One of the earliest examples of conceptual photography is Hippolyte Bayard’s *Self-Portrait as a Drowned Man* (1840), which juxtaposes image and text to confound the viewer with the impossibility that the photographer who has produced the image is in fact a corpse. The punctum in both Bayard and Simonassi’s ‘corpse’ photographs lies in the knowledge that they are playing with the image of death, to question to what extent it can in fact be posed, embodied, or traversed; Simonassi’s images carry a second blow upon recognition that these performed corpses index murders of real people.

**Fig. 9.**
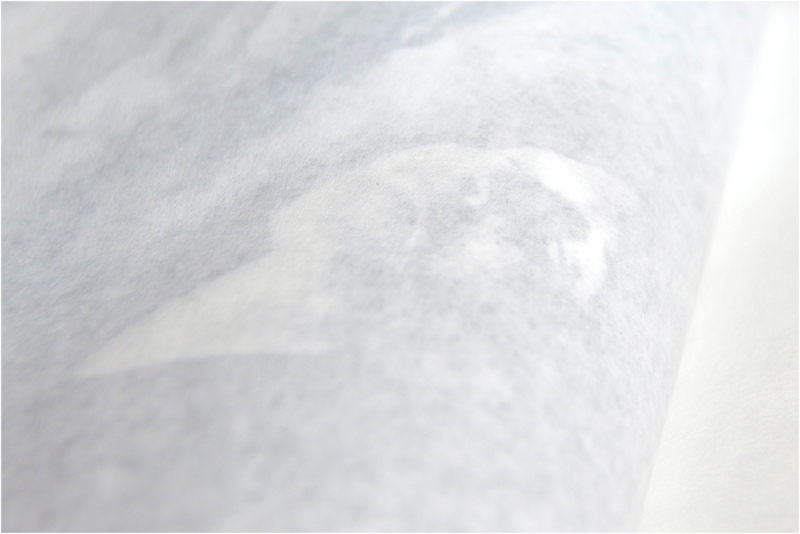
Detail, *Reconstrucción*. the mask-like image of a woman, performed in death by the artist, ‘shrouded’ by the folded paper. Photo: ©Rosana Simonassi.

*Reconstrucción* grew out of Simonassi’s philosophical interest in death as an existential limit, and how different degrees of ‘death’ are structured in society. The artist has a background in philosophy, with particular interest in post-structuralist thinkers such as Michel Foucault. In her previous work, *The Series of 1000 Deaths*, the artist experimented with using her body to present an image of death, much like Bayard before her. However, unlike the early photographer, Simonassi’s corpse-like, performed photographs, printed to scale with her body, were not self-portraits. She described them more like poetry, an effort to explore death as another, ultimate state inhabited by our ever-changing bodies (Figure 9). Anthony Giddens has observed that death ‘is nothing more or less than the moment at which human control over human existence finds an outer limit’, signifying a crisis of modernity and relinquishing our understanding of self, relations with others and even the experience of time.^25^ Simonassi’s work raises questions about the limitations of the image, and imaging death, as she commented, ‘when you’re dead you can’t be a portrait anymore’; the body becomes image.

When both *The Series of 1000 Deaths* and *Reconstrucción* were exhibited, Simonassi told me she found it uncomfortable that people viewed the corpse photographs as portraits. They were interested in the fact that she could be standing in the gallery, whilst being ‘the dead girl’ herself. Like Bayard, her work (perhaps involuntarily) raises the question of whose death is being pictured. In a discussion of ‘decorporealization’ inspired by Bayard’s impossible corpse-portrait, Amelia Jones contemplates our culture’s commitment to viewing the body as both a ‘physical enactment and guarantor of the self’:

Because the photographic portrait embodies a trace of the self (with the mind made visible only through its body-sign), it highlights both the inextricability of body and mind and the fact that we often access the self via its visible form, a form we want to serve as corporeal guarantor of the body.^26^

Contrary to Barthes’ past-tense ‘what was there’-ness, Jones comments that ‘the photographic portrait seems to reaffirm the body’s never-ending “thereness”, its refusal to disappear, its infinite capacity to render up the self in some incontrovertibly “real” way.’

During my encounters with *Reconstrucción*, I had wondered how Simonassi felt as she was posing, embodying a corpse. Her answer was that during a shoot she felt very little. Her attention was consumed by the mechanics of making the photograph. It was through researching each case that she felt proximity to the women, to a point of obsession. This suspension of empathy is mirrored in accounts of those forensic professionals who document horrific scenes of murder. Of crime scene photography, Atėnė Mendelytė writes,

When encountering such imagery, one tends to face strong affective responses such as terror, disgust, sympathy, sadness - one is often salvaging the personhood of the body in question. However, when taking such photographs, one is to take a purely investigative, detached, scientific perspective, completely void of the sympathetic response, treating the body as an object.^27^

**Fig. 10.**
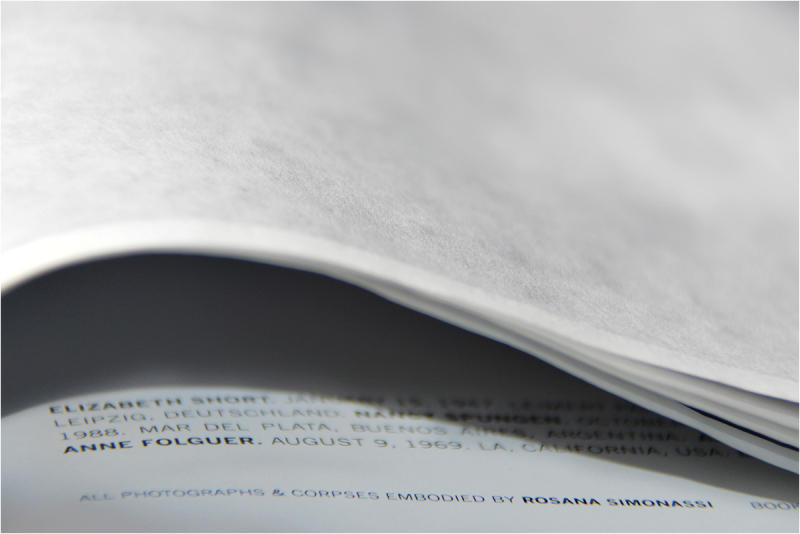
Detail of final page of *Reconstrucción*, containing colophon and victims’ details. Photo: ©Rosana Simonassi.

Photography only defers the affective impact of the task of documenting death. Simonassi’s proximity in staying with these women’s bodies in her research as well as the photographic act appeared to me as a brave gesture of realism, and challenges the media’s commodification of the photographic subject/object. In the photobook, the women in the original source images are identified and remembered in the colophon on the inside back cover (Figures 5 and 10), but until the reader reaches this point, the shrouded ‘bodies’ are also distanced from the viewer through their anonymity. Simonassi’s effort to push viewers/readers to detach the body from a sense of self aligns with Jones’ question of ‘what new kinds of subjects/objects are produced by global capitalist image culture’. Simonassi’s acts of photographing ‘corpses’ are as performative and fleeting as the reader’s encounter with the images in the event of reading the photobook. However, the images are concretely materialised in the photobook format. Instead of ‘decorporealisation’, perhaps in *Reconstrucción* we could talk of ‘recorporealisation’: if Simonassi’s performed corpse-body is a proxy for death more generally, it is a body that could belong to anyone, including the warm body of the reader actively engaged in holding the book. This ethical quandary about attaching subjecthood and the body as object is deepened by the fact that the corpses she is imitating *were* people with identities and a sense of self, as detailed in the final index.

In writing about Aida Chehrehgosha, another artist who photographs ‘painstakingly staged domestic crimes’, Mendelytė questions how boundaries are constructed between art and forensic science.^28^ According to Mendelytė, art photographers restaging crime scenes shape ‘an image which is inherently an index of another index — a death and possibly a crime — which by its nature attempts to eliminate its own mediation and become indexical in a singular sense.’^29^ Simonassi’s mediation, meaning the material interventions in printing and exhibiting her re-staged images, poses obstacles to this elimination. Mendelytė continues, ‘what appears to be the only marker of a certain boundary between art and science in this case is the context in which the photographs appear, which is far from being pre-determined or fixed.’^30^ When crime-scene-adjacent imagery travels into the gallery space, in a carefully considered material form, the situation of its encounter alters its status to more philosophical concept than factual document. As John Tagg has noted, what makes a photograph stand for evidence, or bestows the factuality described by Mendelytė, is not some ‘magic’ quality within the photographic process, but rather, is constructed by the discursive system in which that image is produced and operationalised.^31^ In reconstructing these scenes of femicide, Simonassi questions the limit of our knowledge of death, rather than the reality of an image.

To an extent, by consolidating these complex visual and conceptual objectives of the project into one solid, mobile format, the photobook *Reconstrucción* reorganised the artistic project to expand its international circulation and exposure, following conventional aims of art publishing. The 250 copies that make up the edition have circulated freely around the world, distributing the artwork to wider audiences via book fairs, online sales and placement in public library collections. This global distribution provides exposure for Simonassi and her project that would not be possible with the fragile, limited edition of photographic prints, creating chance encounters between readers and her work, such as my own.

Developing the book with Fieiras also offered Simonassi a meaningful opportunity to further its philosophical enquiry and explore a new logic for audience engagement. As seen in the previous section, the specific, bounded form of the photobook underscores the ways in which people can engage with the artwork with greater control and affective potential than looking at images on a screen. Compared with the fragility of the original prints, which would flutter, tear and fade, the bound mass of the book is arguably more solid and lasting. Still, the photobook conveys its own ephemerality, as I described my reading as being ‘over quickly’.

The book’s affecting physical presence complicates the notion of authorship. While it conforms to publishing practice through casting certain individuals in traditional roles such as ‘author/artist’, ‘designer’, and ‘publisher’, their contributions are felt differently by readers. As I have argued, the materiality of *Reconstrucción*, designed by Fieiras, contributes significantly to its conceptual impact. The singular notion of authorship has been repeatedly contested in histories of art, both in terms of the networked social construction of cultural products, and in reassigning authority to readers/viewers as well as writers/artists/makers.^32^ If Fieiras had not approached Simonassi, the book would never have come to be in the world, in the NAL, on that afternoon — or at least, not in this specific configuration. In the reading encounter, with its shrouded pages and encapsulated dirt, I am as affected by the physical design as the images themselves. I am a live body, interacting with a book of photographs, containing bodies, which signify deaths. These slippages destabilise the photobook as an artwork with ambiguous authorship and inconsistent meaning, and instead present it as a format with open-ended potential for interaction.

## Disruptive dirt: situating a ‘sticky’ photobook

Few materials are as destabilising and non-conformist as dirt. Mary Douglas has called dirt ‘matter out of place’, there are few places dirt is less welcome than museums, which aim to maintain the optimum physical conservation of their objects, as well as accurate classification of everything within in. But dirt is hard to classify: it entails many kinds of dust, grime, mess, decomposing fibres or skin, as well as discourse that is considered amoral or salacious — ‘dish the dirt’, ‘dirty liar’ – in short, things that do not have a place in the sanitised structure of ordinary society.^33^

In *Reconstrucción*, however, dirt is a fundamental part of its design. Books are packaged with light, dusty, dirty powder sprinkled between the covers, much like the stuff of the various floors upon which some victims might have been found or buried in. Upon opening, this would scatter and stick to you: as Jennifer Reeves put it, ‘you yourself become dirty, by handling the book you become complicit’. For most people who purchase the book, the dirt would initially stick to hands, clothes, surfaces, then eventually disperse and dissipate.

When I asked Simonassi about the dirt, she quoted Fieiras, whose idea it was, saying she wanted to trouble the collectors and the bookshelf. This troublesome spirit echoes Frances Stracey’s analysis of the sandpaper covers of Situationist publication *Mémoires*: although the coarse bindings protected the books’ contents, they served as a literal irritation that abraded their bookcase neighbours

to disrupt conventional forms of storage by refusing to be a passive object of contemplation and to become instead an object that grates those trying to handle it. In turn, its own contingency and wearing away would be registered by its rough surfaces being smoothed down over time.^34^

This strategically ambiguous archival object conserves the pre-history of the Situationist movement, meanwhile functioning ‘as a destructive memory, internally cut up and externally abrasive, within the dominant model of a hierarchical history that the Situationists were contesting.’ The dirt in *Reconstrucción*, too, troubles the dominant models of photobook production and consumption it circulates within. The book’s dirt dirties things nearby to it, and in doing so it diminishes its own material presence, unless the collector never opens the plastic shrink wrap to read it. This is not as outrageous as it may sound: designs involving destruction have become recognised as an economic as well as conceptual device, in the hope that collectors might buy multiple copies. This has gained frequency in photobook publishing, to the extent that Moritz Neumüller’s catalogue for the exhibition *Photobook Phenomenon* playfully presented its contents as unbound signatures contained within a cardboard sleeve that had to be ripped open.^35^ Once opened, the effect of *Reconstrucción*’s dirt has been spent, and the book is no longer ‘intact’, again linking back to conceptual gestures of violation and destruction seen in Lacy’s *Rape is*. In this way, *Reconstrucción* both critiques and potentially benefits from photobook economies that involve editioning as a strategy of artificial scarcity.

The addition of the dirt was accentuated in my encounter with the NAL copy of the book because it had been contained within a custom plastic wallet that encased the cover. These transparent CoLibri covers are standard in library conservation, but in this case, Reeves took care to ensure some dirt was encapsulated and visible. I originally thought the plastic cover was part of the book, like an evidence bag. The NAL copy is then kept in an archival card envelope to protect other books from any escaping dust, attempting to discipline an unruly material. This copy of the book has taken on a new aesthetic resonance through its relation to the institution’s conservation priorities. To meet the book’s material demands also required that it was placed in the special rather than general collection, as the protective folder wouldn’t sit securely on a bookcase. Such material disobedience is shared by other photobooks that are made of their unusual subject matter, including the rubber tyre cover of *Bike kill* (2018), by Julie Glassberg, or the special edition of *Vote No.1* (2015) by Mark Duffy, which is bound in the same plastic corriboard election posters it depicts. These unconventional materials raise red flags for conservators because they can release chemicals as they perish that destabilise the carefully controlled storage environment.

The dirt is ‘sticky’, both in how easily it transfers between surfaces and in the sense that Sara Ahmed uses the term to express how concepts accrue affects and meanings.^36^ As Ahmed explains, things, words and ideas ‘become sticky, saturated with affects, as sites of personal and social tension’ when repeatedly associated with certain contexts.^37^ Dirt, colloquially, is ‘stuck’ to the sordidness and wrongdoing Simonassi wished to express. The dirt enclosed with *Reconstrucción*, materially, is stuck to those would-be voyeurs who bear witness to violence. The dirt is nevertheless ephemeral, much like the fragile newsprint paper of the original print series, that yellows with age.

The sticking point of encountering the dirt in *Reconstrucción* can be further reconceptualised in light of new materialist and posthuman scholarship, which frames our understandings of the world in an ever-performing tangle of organic and non-organic, human and non-human agencies.^38^ Dirt’s close, carbon-based counterparts, dust and soil, have captured the imaginations of scholars across arts and humanities.^39^ Mads Daugbjerg and Christopher Whitehead have described the ‘agentic and mnemonic capacities’ of soil, as a mundane, ever-present, yet complex substance that:

bears a seemingly contradictory set of affective projections: it is both inert and yet agentic; amenable yet sometimes hostile; capacious – for example, in holding bodies – yet expulsive; sometimes conceptualised as a spectral actor or ‘witness’, but also allegedly non-sentient.^40^

The dirt in Daugbjerg and Whitehead’s account is a former battlefield in Gallipoli, where histories of conflict that have physically permeated the ground also support memorialising practices. Descendants of fallen soldiers visit the peaceful forest and sometimes take a receptacle of soil home with them, converting dirt into a powerful keepsake, whilst displacing matter out of one site and into another. Depending on how the dirt is framed and conceptualised, it has powerful agency for connecting the past and the present, and becomes symbolic of greater struggles.

As I contemplate the dirt that accompanies *Reconstrucción*, I imagine multiple envelopes opened by soon-to-be-readers, with multiple emanating clouds of dirt, dispersed across a network of those who purchased the book, offering a dusty, distant, distributed material connection. Dust has likewise been framed as connective matter in art historical enquiry in David Campany’s exhibition and book project *A Handful of Dust*, which navigates a meandering critique of modernity and 20^th^ century image-making from Campany’s point of inspiration, Man Ray’s 1920 photograph *Dust Breeding*: ‘inevitable and unruly, dust is the enemy of the modern order, its repressed other’.^41^ Dust (or, generic mixtures of organic and inorganic particles), is generally something that should be kept *out* of the sterile museum store, providing nourishment for pests.^42^ As a former V&A collections employee, my reaction to the incongruence of encountering this loosely encased material was amplified because I couldn’t separate the artistic affect of the presence of dirt from the learned worry about what might happen if it escaped into the collection at large.

Museums seem like places where time stands still, but despite best conservation intentions to slow material change, matter in museums is not inert. Through my NAL encounter with *Reconstrucción* my thoughts wondered back to the topic of death, imagining this book object housed in a monumental accumulation of slowly decomposing matter. To paraphrase new materialist philosopher Jane Bennett, matter is never inactive; even matter that appears eternal is engaged in a process of slow change when viewed on a different, less human-centred timescale. Considered in this light, what differentiates bodies from books? My living, impermanent body of carbon, sitting at an inanimate table of carbon with greater permanence, but not immune to decay, exchanging dust and skin cells with a book-shaped body of other carbon, comes together for the duration of the encounter into what physicist-philosopher Karen Barad might call a performative, looking, perceiving, intra-acting mass of matter for the duration of the encounter.^43^ To a degree, the book is materially embodied and bounded by the edges of its pages, its head, spine and tail, its CoLibri jacket. However, to follow Barad’s agential realism, when I’m looking at the book, a ‘cut’ must be made about where the boundary of my body ends, and the body of the book begins. Both my body and the book-body are ‘containers’ for an identity that is punctuated at that point in materiality and time, as a ‘material-discursive event’, in which our understanding of both matter and meaning (or, ‘mattering’, to use Barad’s term) are co-constituted.

What this tells us is that there is a degree of performativity in understanding what a photobook is, in the moment of a situated encounter. Within the institutional environment, dirt and photobooks share this common resistance to concise definition. *Reconstrucción* is an example of what Star and Griesemer (1989) term a ‘boundary object’: something whose shifting ontological value is performed according to its enrolment in different activities.^44^ The situated reading event in the NAL, with this copy of the photobook, reveals how museums respond to their ambiguity as objects. Photobooks trouble library knowledge systems, because they negotiate a boundary space between artwork and book, depending on how they are discursively framed by the maker, publisher, or cataloguer. In other environments, photobooks perform different functions: in my conversation with Simonassi, the photobook functioned as a connective tissue that initiated and orientated our interaction; in a book fair, a photobook is a commodity to be promoted and carried home, as purchase or unsold stock.

The conceptual bounding of photobooks in the NAL discourages this fluidity. By the time the photobook enters the collection, it has been measured and numbered with a shelf mark that distinguishes its material requirements, and a catalogue record that accessions the book into the special versus general collections. It has been fixed into place like the dirt in the CoLibri cover. This shelf mark has an impact on where and how it is sited within the library storage, as well as how readers are permitted to access and handle the book in the reading room. Once catalogued, the library record contains a few identifying details, such as the author, publisher, title, date, and blurb, usually taken from the internet. Where known, Reeves also credits designer (Fieiras), binder (Isabel Zambelli), printers, paper stocks and other actors contributing to the book’s production, which goes some way to acknowledge the co-productive nature of making photobooks.

Photobooks that enter libraries are singular copies of a larger edition, a distributed whole, so many library systems link their records to a parent record on a system like WorldCat. This networks the NAL copy to a sense of *Reconstrucción* as an ideal book-concept, that exists on some higher Platonic plane than the individual, fallible physical copies. This cataloguing makes an ambiguous book like *Reconstrucción* into something known and singular. It disciplines the object within a pre-determined set of characteristics that reflect its most normative properties and marginalise other potential narratives.^45^ The WorldCat record links *Reconstrucción* to subject headings pulled from other databases including the Art and Architecture Thesaurus (run by Getty) and Library of Congress Subject Headings, which alongside generic key terms such as photobook, also list:

– Death in art

– Female nude in art

– Photography Political aspects

– Photography of women

– Sexual abuse victims

– Violence in art

– Violence in mass media

– Women in art

These headings are not inaccurate, but they essentialise the nuanced reader engagement this photographic object prompts, and the intersectional complexity of issues of gender, representation and violence.

Framing Simonassi’s book under the heading of ‘sexual abuse victims’ impacts how readers interpret the real-world femicide indexed by *Reconstrucción*. Emphasising ‘women’ and not their male killers, who are, where known, detailed in the book’s colophon, feels politicised considering statistics from the UK Femicide Census 2020 that a woman is killed by a man every three days. Public institutions have failed to protect women from violence by known and trusted men. Political decision-making and media portrayals to violence against white women and women of colour vary drastically. This is of course not limited to the UK and Argentina, but a universal issue resulting from entrenched patriarchy, which is entangled with the imperialist agendas of the 18^th^ and 19^th^ centuries in which contemporary museum and library systems are rooted.

While collections need keywords and labels so their parts can be located, this categorisation is always politicised. The original epistemological project of museums was to organise and reduce complex networks of objects and histories into singular, often arbitrary categories, often in the name of documentation, but which affirmed certain hegemonic narratives.^46^ *Reconstrucción’s* rejection of this is not new: in the wider history of artists’ books and printed matter, Suzanne Lacy and Womenspace, Lucy Lippard, Joan Lyons and other feminist book artists of the 70s, Carolee Schneeman, the Situationist movement, have leveraged this ontological ambiguity to create space for marginalised politics. While this potential gives the contemporary photobook a capacity to express subaltern experiences, it also maintains their ‘problem’ status for traditional practices of cataloguing and display.

Feminist scholars of archival practices and digital humanities have long critiqued the failures of archives to account for cultural nuance and social difference.^47^ Accounts such as Lozana Rossenova and Karen Di Franco’s feminist approach to modelling archival data around different iterations of Carolee Schneeman’s artist’s book *Parts of a Body House Book* (1972) demonstrate that new organisational frameworks are possible to better accommodate polyvalence and relationality.^48^ It is conceivable that the immense global institutionalisation of categories by systems like WorldCat, and their resulting classificatory co-ordinates could be constructed with alternative social agendas that better accommodate fluidity and ambiguity. For example, in the Sitterwerk Kunstbibliothek in Switzerland, the art library has given its records Radio Frequency Identification tags instead of pressmarks, so they may be found anywhere within the library’s shelving and can be grouped according to the serendipitous research interests of their users, rather than keywords, which predispose viewer-readers towards certain judgements about visual media.^49^

**Fig. 11.**
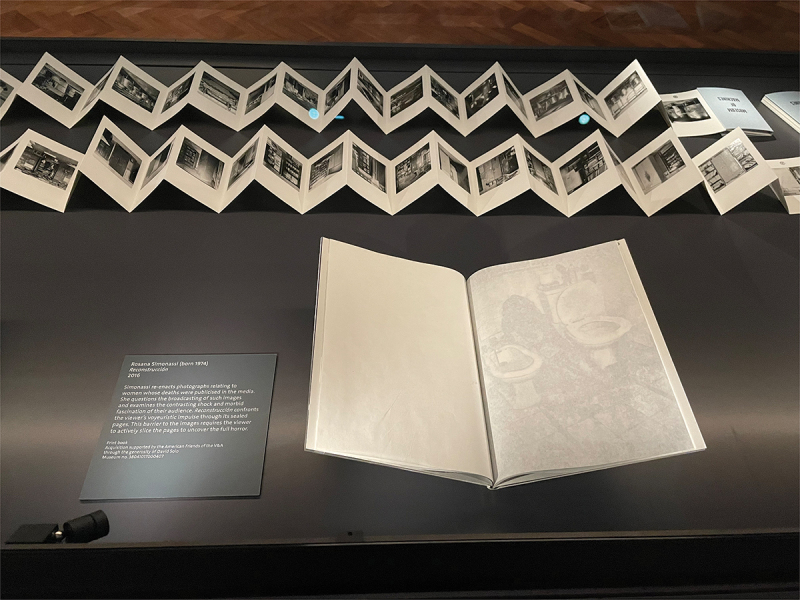
*Reconstrucción* in a vitrine in the V&A photography centre, October 2022. Photo: Briony Carlin.

My NAL encounter with *Reconstrucción* conceptualises how photobooks are more generally understood because they are objects that are made in multiple and distributed globally, and there exist culturally coded modes of photobook production in different parts of the world. Simonassi touched on this in describing to me the difference between the established market for photobooks that exists in Europe, compared to fewer opportunities and audiences in Argentina. Even with the same copy, I can trace how encounters have added different discursive layers to my understanding of *Reconstrucción*, as well as the shifting status of the photobook more generally. More recently, I saw *Reconstrucción* on display in a vitrine in the V&A Photography Centre, sitting upon a custom-made book cradle, with an accompanying interpretive text panel (Figure 11). Although the increasing institutional presence of photobooks in exhibitions as well as collections demonstrates the growing economic and popular interest in the medium, the glass case formed a barrier that prevented the complex feelings of complicity or voyeurism activated by handling the object, once again disciplining engagement between the bodies of reader, maker and subject.

## Conclusion

The situated encounter with a photobook both confirms its relation to established art publishing formats, practices of circulation and institutional consumption, meanwhile destabilising them. *Reconstrucción* is a provocative object that restricts conventional ways of looking, and invites other ways of seeing, touching and feeling.

This article has advanced a complex analysis of three components of *Reconstrucción*, folds, death and dirt. The case study has exemplified ways in which photobooks typically function to present and circulate a body of work and support creative collaboration between stakeholders, whilst destabilising the notion of authorship. By situating the photobook in relation to Simonassi’s wider artistic enquiry, the article has demonstrated how photobooks can be connected to lineages of art, publishing and photography histories.

Through attending to *Reconstrucción’s* complicated material presence as an object, I have demonstrated how materiality, embodied reading experiences and institutional systems of classification have exerted power over the bodies of women — those indexed by the book, and that of the reader — which remains a necessary task in a persistently patriarchal media landscape. At the same time, it has revealed insights into the affective potentials of the photobook medium and offered critique of a primary site of photobook production and consumption. Finally, this article has revealed tensions in how photobooks are encountered in institutional spaces, whether through their databases or barriers to cultural access in their reading rooms, both of which make meanings about the ontological understanding of this individual photobook, and the medium more generally.

## Notes

1. As described on the website for *Editorial Chaco*.

2. Colberg, *Understanding Photobooks*; Östlind, *Published;* Johnston, *Photobooks &*; Julià and Cataldo, *Photography Bound*; and Lederman, Yatskevich and Lang, *How We See.*

3. Pol and Vorstermans, *Future Books*, 22.

4. This has also been noted by Matt Johnston in *Photobooks &;*, 13, 19; and for example, Parr and Badger, *The Photobook: A History;* Roth, *The book of 101 books*; Fernández, *Fotografia publica*; and Mattie Boom, *Photography Between Covers.*

5. Edwards, *The Photobook World*, 12.

6. See Julià and Cataldo, *Photography Bound*, 22

7. Brown, “Intimate Archive,” 3.

8. Edwards, *The Photobook World.*

9. As also noted by: Liberty, Reynolds, & Solo, “A New Manifesto For Book Art Criticism; Carlin, *Bindings, Boundaries and Cuts …;* Johnston, “A New Materiality,” *Over Journal*, issue 3, 2022.

10. Wetherell, *Affect and emotion*; Massumi, “The Autonomy of Affect,” 88.

11. See Roland Barthes’ discussion of “writerly” and “readerly” texts in *The Pleasure of the Text*; also the postmodern condition of authorship in “The Death of the Author,” 142–149.

12. Bond, “The photobook as event,” see *The Photobook World*; see also Star and Griesemer, “Institutional Ecology” for discussion of boundary objects.

13. Manning, *Minor Gesture.*

14. See Haraway. “Situated Knowledges”; and “A Game of Cat’s Cradle”.

15. Badovinac, *Unannounced Voices*.

16. There is a groundswell of autoethnographic and autotheoretical modes of research that practice what Chadwick calls “epistemic generosity” in “The Question of Feminist Critique”; modes that challenge antagonistic and interrogative approaches to data and experience, instead drawing on other styles of critique as caring and affirming. Likewise; this can be constructed through personal reflection on research material, and is importantly sited in the researcher’s subjectivity. See also, Zwartjes, “Autotheory As Rebellion”; Grocott, Sumartojo et al., “Wandering Feasts”; Adams, Holman Jones and Ellis. *Handbook of Autoethnography*; Harris and Holman Jones. *Affective Movements, Methods and Pedagogies.*

17. Jennifer Reeves is now Resource Sharing Librarian at the NAL. She has been instrumental in the displays of photobooks in the Photography Centre galleries as well as the new Royal Photographic Society Library at the V&A South Kensington.

18. Several writings on artists’ books have reflected on the book as a “container” or “cage,” for example Tate Shaw, “Reading as Prowling, Book as Cage,” *Blurred Library*.

19. This discourse relates to how museums create architectural and biopolitical epistemologies that predispose and discipline behaviours in visitors through the spatial, visual, rhetorical and monumental features of their buildings and displays, see: Duncan, *Civilizing Rituals*; also: Rees Leahy, *Museum Bodies;* Rounds, “Doing Identity Work in Museums,”.

20. Carlin, “Returning to Another Black Darkness … ,” 57–8; see also, Sumartojo and Pink, *Atmospheres*.

21. There is a rich body of recent literature that can be linked to 1990s New Museology, i.e. Bennett, *The birth of the museum;* see Brett Shaindlin, “Reading Museum Exhibits”; Chynoweth, *Museums and the Working Class*; Robert, “Getting Intersectional in Museums”.

22. Robinson, *Crime Scene Photography*.

23. “The Green Wave,” Amnesty International.

24. Paraphrased from Barthes. *Camera Lucida*, 95–6.

25. Giddens, *Modernity and Self-Identity*, 162.

26. Jones, “Decorporealization,” 134.

27. Mendelytė, “Creative and Crime Scene Photography,” 174.

28. Ibid., 168.

29. Ibid., 176.

30. Ibid., 172.

31. Tagg, *Burden of Representation*, 4.

32. For further discussions of the social construction of art, see Becker, *Art Worlds;* and Bourdieu, *The Field of Cultural Production*. Other scholars have long sited the production of “art” in the act of interpretation and embodied meaning-making, for example Dewey, *Art as experience*; Sontag, *Against Interpretation*.

33. Here I’m drawing a parallel between Douglas’ notion of “purity” (see *Purity and Danger)* and museum neutrality/conservatism.

34. Stracey, *Constructed Situations*, 24.

35. In one museum collection, this was done carefully with a scalpel, so the fastening remained attached, see Carlin, *Bindings, Boundaries and Cuts …* , 202.

36. Ahmed, “Multiculturalism,” 126.

37. Ahmed’s use of this term initially referred to how commonplace words such as “swamped” and “flooded” adhered to negative, emotive signification in political discourse around asylum seekers in the early 2000s, in a wider discussion of the relation between affective, sensory description and intersectional politics of language. See also, Ahmed, “Affective Economies,” 120.

38. Barad, *Meeting the Universe Halfway;* Bennett, *Vibrant matter;* Braidotti, *The Posthuman*.

39. Puig de la Bellacasa and Tsing have individually written of the agency of soil in enabling sustained, connected, and at times, “caring” ecologies among worms, mushrooms and agricultural industry.

40. Daugbjerg and Whitehead, “The grounds of Gallipoli,” 3.

41. Campany, *Handful of Dust.*

42. Shah et al. “When the Dust Settles”; Uring, Chabas, and Alfaro, “Dust deposition”.

43. Barad, *Meeting the Universe Halfway.*

44. Star and Griesemer, “Institutional Ecology,” 393.

45. Zepeda, “Queering the Archive”; Kim, “Building Pleasure”.

46. Whitehead, *Museums and the Construction of Disciplines*, 40.

47. See D’Ignazio and Klein, Data Feminism; Caswell, Urgent Archives; Morra, *Moving Archives*.

48. Rossenova and Di Franco, “Iterative Pasts”.

49. See essay “Dynamic Order and the Kunstbibliothek” on the Stiftung Sitterwerk website.
